# First Draft Genome Sequence of Xanthomonas axonopodis pv. eucalyptorum, Causal Agent of Bacterial Leaf Blight on Eucalypt

**DOI:** 10.1128/MRA.00120-19

**Published:** 2019-04-18

**Authors:** Yane F. Neves, Samuel A. Santos, Lúcio M. S. Guimarães, Pedro M. P. Vidigal, Jorge L. Badel, Poliane Alfenas-Zerbini, Reginaldo G. Mafia, Acelino C. Alfenas

**Affiliations:** a Laboratory of Molecular Phytobacteriology, Department of Plant Pathology, Universidade Federal de Viçosa, Viçosa, Minas Gerais, Brazil; b Laboratory of Forest Pathology, Department of Plant Pathology/Instituto de Biotecnologia Aplicada a Agropecuária (BIOAGRO), Universidade Federal de Viçosa, Viçosa, Minas Gerais, Brazil; c Núcleo de Análise de Biomoléculas (NuBioMol), Centro de Ciências Biológicas, Universidade Federal de Viçosa, Viçosa, Minas Gerais, Brazil; d Department of Microbiology, Instituto de Biotecnologia Aplicada a Agropecuária (BIOAGRO), Universidade Federal de Viçosa, Viçosa, Minas Gerais, Brazil; e Centro de Tecnologia, Fibria Celulose S.A., Aracruz, Espírito Santo, Brazil; University of Delaware

## Abstract

Here, we report the annotated draft genome sequence of Xanthomonas axonopodis pv. eucalyptorum pathotype strain LPF602 (synonym Xanthomonas axonopodis BSC45a), isolated from eucalypt leaves showing bacterial blight symptoms in Brazil. The availability of these genomic data will help improve the understanding of the evolution and molecular mechanisms involved in the pathogenesis of this microorganism.

## ANNOUNCEMENT

Bacterial leaf blight (BLB) is an important disease of eucalypt in the field under a high rainfall regime and under nursery conditions (1–3). The losses caused by BLB in Brazilian nurseries exceeded 7.0 million dollars between 2003 and 2008 (2). Characteristic symptoms of BLB include foliar deformation and water-soaked lesions, which evolve to necrosis that can form central perforations and promote intense defoliation (1–3). In 2008, a detailed study with strains collected from several different geographic areas reported that Xanthomonas axonopodis is the most frequent causal agent of BLB on Eucalyptus spp. in Brazil (1).

Recently, the new pathovar *X. axonopodis* pv. eucalyptorum was reported to be the causal agent of BLB on *Eucalyptus* spp. and *Corymbia* spp. based on polyphasic taxonomy in association with multilocus sequence analysis, and LPF602 was designated a pathotype strain (4). Strain LPF602 was isolated from an infected 2-year-old Eucalyptus grandis × Eucalyptus urophylla hybrid clone in a field located in Texeiras de Freitas, Bahia, Brazil, and was originally referred to as *X. axonopodis* BSC47a (1). Because of the recent description of this pathosystem, there is still no information available on the genetic diversity and evolution of *X. axonopodis* pv. eucalyptorum. Hence, genome sequences of *X. axonopodis* pv. eucalyptorum will provide important information on these and other aspects of its biology, including insights into the molecular mechanisms underlying its pathogenicity. Here, we make available the draft genome sequence of *X. axonopodis* pv. eucalyptorum pathotype strain LPF602.

Strain LPF602 (1, 4) was grown on solid 523 medium (5) at 28°C for 48 h. DNA was extracted from pooled colonies on the plate using the Wizard genomic DNA purification kit (Promega, Madison, WI, USA) following the manufacturer’s instructions. Library preparation with the NEBNext Ultra II DNA library prep kit (New England Biolabs, Ipswich, MA, USA) with sample purification beads optimized for 350-bp size-selected DNA fragments and sequencing using the NovaSeq 6000 Illumina platform with 2 × 150-bp paired-end reads were provided by GeneOne Biotechnology (Bangu, Rio de Janeiro, Brazil). A total of 5,198,692 paired-end reads were obtained. Low-quality reads were trimmed and filtered by AfterQC using default parameters (6). High-quality reads were *de novo* assembled into scaffolds using SPAdes version 3.10.1 testing different k-mers (from 23 to 123) (7). The NCBI Prokaryotic Genome Annotation Pipeline (PGAP) (8) was used for gene prediction and annotation, and Benchmarking Universal Single-Copy Orthologs (BUSCO) was used to estimate the completeness of the assembled genome (9), in both cases using default parameters.

The 5.2-Mb *X. axonopodis* pv. eucalyptorum genome was assembled into 62 scaffolds with sizes ranging from 1,000 nucleotides (nt) to 637 kb, an average scaffold length of 83 kb, an *N*_50_ value of 244 kb, and an average coverage of 50-fold. The genome has a G+C content of 64.6% and a total of 4,198 predicted genes. The completeness assessment of the genome by BUSCO identified 100% complete and single-copy gene groups and no duplicated, missed, or fragmented gene groups specific for bacteria, which indicates that the assembled genome covers most of the coding regions.

This is the first draft genome sequence of *X. axonopodis* pv. eucalyptorum, an important bacterium pathogenic to eucalypt. The access to this information will allow future studies aimed at understanding the biology of this phytopathogen by comparison of its genome with those of other *X. axonopodis* pv. eucalyptorum strains and *X. axonopodis* pathovars.

### Data availability.

This whole-genome shotgun project has been deposited at DDBJ/ENA/GenBank under the accession number RWJW00000000. The version described in this paper is the first version, RWJW01000000. The raw data were also deposited in the Sequence Read Archive under accession number SRR8303373.
